# Personalisation of Molecular Radiotherapy through Optimisation of Theragnostics

**DOI:** 10.3390/jpm10040174

**Published:** 2020-10-16

**Authors:** LauraMay Davis, April-Louise Smith, Matthew D. Aldridge, Jack Foulkes, Connie Peet, Simon Wan, Jennifer E. Gains, Jamshed B. Bomanji, Mark N. Gaze

**Affiliations:** 1 Department of Nuclear Medicine, University College London Hospitals NHS Foundation Trust, 235 Euston Road, London NW1 2BU, UK; lauramaydavis@nhs.net (L.D.); april-louise.smith@nhs.net (A.-L.S.); matthewaldridge@nhs.net (M.D.A.); jamshed.bomanji@nhs.net (J.B.B.); 2 Department of Oncology, University College London Hospitals NHS Foundation Trust, 250 Euston Road, London NW1 2PG, UK; jack.foulkes@nhs.net (J.F.); connie.peet@nhs.net (C.P.); mwan@nhs.net (S.W.); jenny.gains@nhs.net (J.E.G.)

**Keywords:** dosimetry, imaging biomarkers, molecular radiotherapy, organs at risk, personalised dose administration, radionuclide therapy, response assessment, theragnostics

## Abstract

Molecular radiotherapy, or targeted radionuclide therapy, uses systemically administered drugs bearing a suitable radioactive isotope, typically a beta emitter. These are delivered via metabolic or other physiological pathways to cancer cells in greater concentrations than to normal tissues. The absorbed radiation dose in tumour deposits causes chromosomal damage and cell death. A partner radiopharmaceutical, most commonly the same vector labelled with a different radioactive atom, with emissions suitable for gamma camera or positron emission tomography imaging, is used to select patients for treatment and to assess response. The use of these pairs of radio-labelled drugs, one optimised for therapy, the other for diagnostic purposes, is referred to as *theragnostics*. Theragnostics is increasingly moving away from a fixed number of defined activity administrations, to a much more individualised or personalised approach, with the aim of improving treatment outcomes, and minimising toxicity. There is, however, still significant scope for further progress in that direction. The main tools for personalisation are the following: imaging biomarkers for better patient selection; predictive and post-therapy dosimetry to maximise the radiation dose to the tumour while keeping organs at risk within tolerance limits; imaging for assessment of treatment response; individualised decision making and communication about radiation protection, adjustments for toxicity, inpatient and outpatient care.

## 1. Introduction

Radiotherapy is the use of ionising radiation to treat medical conditions, almost always malignant disease. Molecular radiotherapy, sometimes called radionuclide therapy, is a treatment at the interface of radiation oncology and nuclear medicine. It is defined as the systemic administration, for example intravenously or orally, of radiopharmaceuticals, or drugs incorporating unsealed radioactive sources, to target cancer cells. These radioactive drugs are accumulated through physiological processes and retained in tumour tissue to a greater extent than in healthy organs, leading to the localised delivery of substantial doses of ionising radiation to cancer cells, resulting in single and double strand breaks in nuclear DNA (deoxyribo-nucleic acid), and cell death. Corresponding radiopharmaceuticals, bearing radioactive isotopes which have been selected because their emissions are optimal for visualisation by either gamma camera or PET (positron emission tomography), are used for molecular imaging. This demonstrates the extent and location of disease within the body, which may be suitable for treatment with molecular radiotherapy, and can also be used for response assessment subsequently.

Theragnostics, sometimes called theranostics, is a neologism based on the words therapy and diagnostics, bringing together the molecular imaging used for diagnostic purposes with the molecular radiotherapy used for treatment as a single concept. For each clinical purpose, there is a theragnostic pair of radiopharmaceuticals, one for imaging, one for therapy. In some cases, for example when there is a choice between gamma camera and PET imaging, there may be more than a pair of agents available. Examples of theragnostic pairs in use are shown in Table 1.

The earliest clinical use of theragnostics was over three quarters of a century ago, with the introduction of radioactive iodine treatment for thyroid cancer [1]. For many years, standard administered activities, and a standardised number of treatment courses, have been used. The success of some treatments has been a barrier to attempts to improve outcomes through the personalisation of molecular radiotherapy.

One example of this is the use of ^177^Lu DOTATATE treatment in the management of well-differentiated metastatic mid-gut neuroendocrine cancers which express somatostatin receptors. In the NETTER-1 trial, patients were randomised to receive either a standard fixed administered activity protocol comprising four administrations at 8-week intervals of 7.4 GBq ^177^Lu DOTATATE, or only long-acting octreotide repeatable injections. Molecular radiotherapy was shown dramatically to improve outcomes: the event-free survival at 20 months in the experimental arm was 65.2%, compared with only 10.8% in the control arm [2]. In addition, better quality of life indices were observed in those receiving ^177^Lu DOTATATE [3]. While this trial clearly shows the benefit of this standardised approach to molecular radiotherapy over the comparator treatment, not every patient did well. It is known that patients with a target lesion greater than 3 cm diameter did less well than those with only smaller lesions, regardless of whether the overall tumour burden was low, medium or high [4]. It is also known that re-treatment with the same agent after disease progression is safe and worthwhile [5].

These observations prompt us to wonder whether a more personalised approach to initial ^177^Lu DOTATATE therapy in patients with metastatic neuro-endocrine cancers, rather than the rigid schedule of only four fixed activity administrations, as recommended in the United Kingdom’s NHS (National Health Service) by NICE (National Institute for Health and Care Excellence) [6], might lead to better initial outcomes. For example, as renal damage is the principal dose-limiting toxicity, could the administered activity be titrated against the measured renal radiation dose? This would allow a maximum tolerated administered activity to be given, which might be substantially greater than the four administrations of 7.4 GBq. Or, as we know those with tumours larger than 3 cm diameter fare worse, possibly these patients could be selected for dose escalation. Further research to optimise outcomes based on a more personalised approach is clearly indicated.

Historically, molecular radiotherapy employed very standard treatment regimens. However, as we learn more, and as new technology and tracers become available, there is a gradual trend towards increasing the personalisation of care [7]. Some typical “dosing” (administered activity) schedules in current use and the dosimetry routinely performed are described in Table 2. This article reviews some ways in which the theragnostic approach has been be personalised, or could be individualised further. Examples are given of the evolution of treatments over time, and of clinical trials which seek to increase the evidence base so that molecular radiotherapy can be used in a more intelligent and nuanced way than has been the case in the past. The nuclear medicine and oncological community must move away from a stochastic evaluation of the success of a treatment—it either works or it does not—and find ways to optimise the patient experience and clinical outcomes.

## 2. Imaging Biomarkers

### 2.1. Differentiated Thyroid Cancer

Typically, in the postoperative management of differentiated thyroid cancer, radioactive iodine ablation is used without prior imaging, as iodine uptake by residual normal thyroid cells, and cells of differentiated thyroid cancers of follicular cell origin, is universal. The British Thyroid Association Guidelines for the Management of Thyroid Cancer do not recommend any molecular imaging prior to radioactive iodine remnant ablation [8]. However, there is some evidence that prior ^123^I scanning may lead to a change in management in some cases, making this a true personalised theragnostic approach [9]. Prior ^131^I scanning is an alternative, and concerns that this may lead to a greater risk of ‘stunning’ compared with ^123^I scanning, and therefore to the reduced therapeutic efficacy of the ablation administration, appear unfounded [10]. ^18^F FDG (fluorodeoxyglucose) PET CT (computed tomography) is an alternative diagnostic imaging technique which can be used prior to remnant for risk stratification, to facilitate a more personalised approach [11].

### 2.2. Neuroblastoma

Molecular radiotherapy is possibly most valuable where disease is widespread in the body, so not amenable to a localised approach to treatment such as surgery or external beam radiotherapy. In addition, there is a requirement for the cancer cells to express one or more physiological pathway which can be exploited by a molecular vector to allow the accumulation of radio-isotope in the tumour. Ideally, physiological accumulation will be less marked in non-target tissues. It is also beneficial, although not absolutely necessary, if the tumour is relatively radiosensitive.

The childhood cancer neuroblastoma fulfills all these criteria:

- Neuroblastoma cells have been shown to be intrinsically highly radiosensitive, with a limited repair capacity [12];
- The disease is often metastatic to bone and bone marrow, and less commonly to other organs [13];
- Immunohistochemistry demonstrated that neuroblastoma cells express the noradrenaline transporter molecule, responsible for the uptake of mIBG, and the somatostatin receptor, responsible for uptake of somatostatin analogues such as DOTATATE [14].

**Figure 1:**
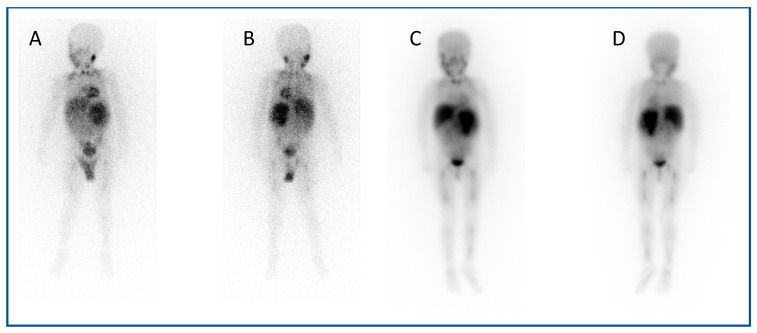
An example of a theragnostic pairing of radiopharmaceuticals in a child with metastatic neuroblastoma. Diagnostic ^123^I-mIBG imaging (**A**) anterior and (**B**) posterior views of whole-body planar scintigraphy demonstrating pathological uptake in a large left sided retroperitoneal mass as well as in metastatic disease in the femora and jaw, as well as physiological uptake in the salivary glands, heart, liver, urinary bladder and (despite blockade) in the thyroid gland. Corresponding (**C**) anterior and (**D**) posterior images following ^131^I-mIBG therapy administration showing uptake of the agent in the tumour and metastases as well as in physiological sites.

^123^I mIBG (*meta*-Iodobenzylguanidine) scintigraphy is the gold standard imaging modality for staging in neuroblastoma (Figure 1A,B), and various semi-quantitative systems have been devised which correlate with prognosis [15,16,17]. Over 90% of patients with neuroblastoma express the noradrenaline transporter molecule and so take up mIBG [18], and scintigraphic demonstration of this is used to select patients for ^131^I mIBG therapy (Figure 1C,D) [19].

The level of somatostatin receptor expression can be gauged on ^68^Ga DOTATATE PET CT, and ^177^Lu DOTATATE can be used for therapy [20,21,22]. While both ^131^I mIBG and ^177^Lu DOTATATE therapy may have a place in the treatment of neuroblastoma, discordance has been shown in the anatomical localisation of disease by somatostatin receptor and noradrenaline transporter molecule imaging [23]. Treatment plans may potentially be more personalised by taking into account the results of both forms of imaging.

### 2.3. Metastatic Prostate Cancer

Theragnostic approaches to the treatment of painful bony metastases commonly seen in prostate cancer were first presented in 1976. A series of patients initially imaged with ^85^Sr, a calcium analogue, for diagnostic purposes reported an analgesic benefit [24]. The subsequent use of ^89^Sr, a beta-emitter, resulted in a lasting improvement in bone pain. This was further evaluated in patients with bone metastases due to both breast and prostate cancer who were initially assessed with strontium-85 bone scintigraphy [25]. Over a 3-month period, 15% reported becoming pain-free, and no patients reported a worsening of symptoms. Additional beta-emitting radiopharmaceuticals, that can be used in the setting of osteoblastic bony metastases, and in mixed lesions, include ^153^Sm lexidronam and ^186^Re etidronate [26]. Bone scintigraphy with ^99m^Tc HDP (hydroxymethylene diphosphonate) should be performed 4 weeks prior to treatment to confirm osteoblastic activity, and therefore the suitability for treatment. Patients require close assessment of haematological and biochemical parameters; marrow suppression may occur, and the appearance of a ‘superscan’ on the ^99m^Tc HDP scintigram suggests significant bone marrow involvement, and in most cases presents a contra-indication to beta-emitter therapy. The administered activities advised by EANM (the European Association for Nuclear Medicine) for ^89^Sr and ^186^Re etidronate are fixed (150 MBq and 1295 MBq, respectively), whilst ^153^Sm lexidronam is weight-based (37 MBq/kg). Re-treatment can be considered in responsive patients with recurrent pain, with a minimum period of 8 weeks for ^153^Sm, 6–8 weeks for ^186^Re and 12 weeks for ^89^Sr. More rigorous dosimetric calculation is rarely required due to the advanced state of the disease at time of treatment and the short duration of survival in this population.

^223^Ra presents an alternative treatment for proven osteoblastic bony metastases [27]. ^223^Ra emits alpha radiation, which has a far shorter mean free path than the beta particles of ^89^Sr, ^153^Sm and ^186^Re, with energy deposition within a far shorter range. As a result, ^223^Ra dichloride, once taken up as a calcium analogue by osteoblasts at the site of osteoblastic bony metastases, has a significantly shorter range and higher linear energy transfer than beta-emitter radiopharmaceuticals, and the damage to adjacent normal tissue is minimised, with less myelotoxocity [28]. The ALSYMPCA trial demonstrated an increased time to first symptomatic skeletal event in ^223^Ra compared to placebo [29]. ^223^Ra is given as a fixed administration according to weight, although it does emit a few 82keV photons, potentially allowing for post-therapeutic imaging and personalised dosimetry in future, allowing better control of haematological toxicities. Furthermore, adaptive treatment planning, in which a low activity of ^223^Ra is given prior to the first therapy to assess biodistribution, and thus to allow the planning of therapy to minimise gut, bone marrow and endosteal absorbed dose, has also been studied [30].

As ^223^Ra, ^89^Sr, ^153^Sa and ^186^Re exert their effects by acting as calcimimetics, being taken up by osteoblasts at the site of proven osteoblastic bony metastases, and they only target malignant cells indirectly, accumulating at the site of osteoblastic activity and irradiating the tumour as a bystander effect. One limitation of this is that any bony metastatic deposits that have not induced an osteoblastic response will, therefore, not accumulate the radiopharmaceutical.

**Figure 2:**
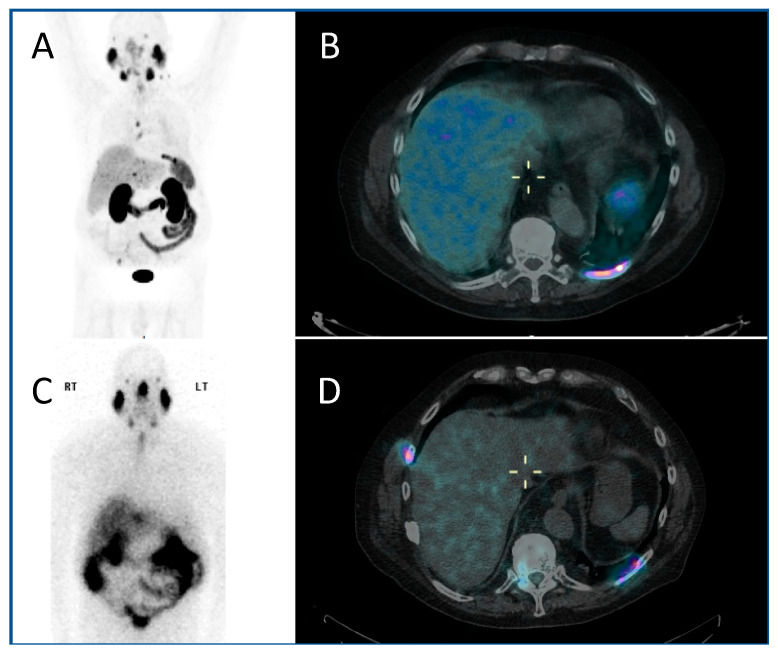
A PMSA-based theragnostic approach to metastatic prostate cancer. (**A**) Anterior view of maximum intensity projection, and (**B**) axial view of ^68^Ga-PMSA PET CT (positron emission tomography, computed tomography) demonstrating left posterior rib metastasis. Then, following ^177^Lu PMSA administration, (**C**) anterior view planar scintigraphy and (**D**) SPECT CT (single photon emission computed tomography, computed tomography) imaging. While these show similar appearances, post-therapy imaging has a lower resolution as the techniques are different.

The use of PSMA (prostate-specific membrane antigen) targeted radiopharmaceuticals overcomes this, offering a more personalised treatment, targeting as it does the PSMA receptor on the cell surface of prostate cancer cells directly. Prostate-specific membrane antigen is a 750 amino acid type II transmembrane glycoprotein. It is expressed by most epithelial cells, but over-expressed (up to 1000-fold) by the majority of prostate cancer cells. Diagnostic PET imaging with ^68^Ga PSMA exploits the internalisation undergone by the receptor, allowing radioisotopes to be concentrated within the cell, having bound to the external component of the PSMA (Figure 2A,B). Uptake is also seen in the salivary glands, kidney and proximal small intestine. There is a correlation between the higher Gleason score of prostate cancers, castrate-resistance and PSMA expression, making the PSMA receptor an optimal target for theragnostic development [31].

The PSMA-based radiopharmaceuticals developed for therapy include ^131^1, ^90^Y and ^177^Lu. The side effects of all forms of PSMA-based radiopharmaceutical included dose to salivary glands and kidneys. ^177^Lu is a beta-emitter, with a shorter range than ^90^Y (1.5 mm ^177^Lu; 12 mm ^90^Y), giving it a more desirable side-effect profile. There is a good correlation between ^68^Ga PSMA and ^177^Lu PSMA distribution, allowing for pre-therapeutic dosimetric calculation to be performed in order to minimise toxicity (Figure 2C,D) [32]. ^177^Lu emits co-incident gamma emission, allowing post-therapeutic imaging to both confirm uptake, and allowing dosimetric calculation for subsequent therapeutic administrations [33]. ^177^Lu DOTA-PSMA-617 is the most extensively investigated radiopharmaceutical of this class, with its efficacy demonstrated in phase II randomised trials which showed a biochemical response in two thirds of patients. Phase III trials—TheraP and VISION—are currently underway, with completion expected in 2021 [34,35].

### 2.4. Liver Metastases

**Figure 3:**
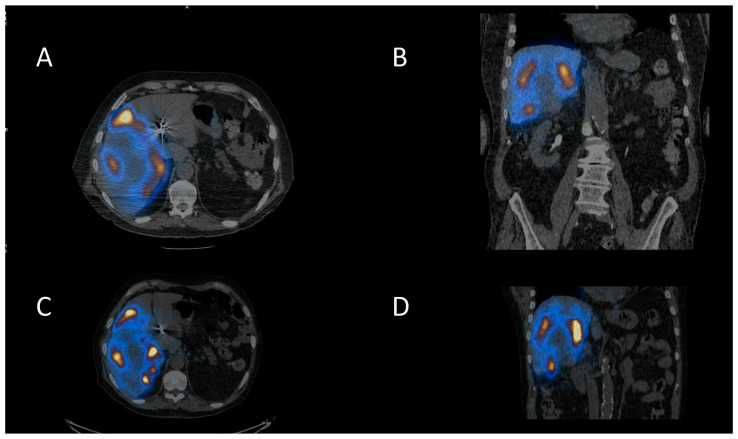
Selective internal radiotherapy with radiolabelled microspheres for liver metastases. (**A**) Axial and (**B**) coronal fused SPECT CT images of ^99m^Tc-MAA (macroaggregated albumin) pre-treatment imaging for dose calculation and treatment verification. Corresponding (**C**) axial and (**D**) coronal post-therapy PET CT images following therapy with ^90^Y-microspheres.

Selective Internal Radiotherapy (SIRT) is a treatment using radiolabelled microspheres to treat liver tumours with indications including hepatocellular carcinoma (HCC) and liver metastases from colorectal cancer (mCRC) [36]. Benefits have been seen for the treatment of liver metastases for some other primary cancers as well [37]. Radiobiologically guided dosing strategies are possible [38]. Patients undergo a hepatic angiogram to perform embolisation to prevent shunting to extrahepatic areas and to administer ^99m^Tc-MAA (macroaggregated albumin) for imaging prior to treatment. The treatment consists of ^90^Y-microspheres, which are delivered through the same hepatic route to deliver the dose directly to the tumours. The ^90^Y-microspheres remain in situ, permanently irradiating the tumour sites until the radioactivity physically decays away. The work up imaging allows dose calculation for personalised treatment and allows the identification of shunting. There are two types of ^90^Y-microspheres: resin spheres at 20–60 µm diameter and glass spheres 20–30 µm. ^90^Y is a predominantly beta-emitting radioisotope, so imaging can be challenging. Bremsstrahlung imaging or PET imaging is required to image post therapy. A workup with ^99m^Tc-MAA and PET imaging of the ^90^Y-microspheres is shown in Figure 3.

^166^Ho–microspheres are a newer product on the market, which are a gamma-emitters and have paramagnetic properties allowing for SPECT and MR imaging. The product also has the option of low-dose ^166^Ho-microspheres are for the workup to more accurately mimic the treatment, as they use the microspheres themselves for the pre-therapy imaging. [39]

## 3. Dosimetry

It needs to be recognised that there is a big difference between the amount of a radiopharmaceutical given to a patient, sometimes colloquially called the ‘dose’, and the amount of radiation absorbed by the tumour. It is preferable to refer to the former as the administered activity (measured in GBq), and restrict the use of the word dose to the absorbed radiation dose to the whole body or tumour (measured in Gy).

The administered activity is usually known quite accurately. It is often a fixed amount, for example 1.1 GBq or 3.5 GBq for thyroid remnant ablation with ^131^I [40], or 7.4 GBq for the treatment of neuroendocrine cancers with ^177^Lu DOTATATE [2]. In other cases the administered activity may be scaled to weight or body surface area. For example, in a study of the escalation of the administered activity of ^131^I mIBG for neuroblastoma, the prescribed administered activities were 444, 555 and 666 MBq/kg [41].

What is often unknown, because it is not measured in most studies, is the resulting whole-body or tumour dose. However, in this study, detailed dosimetry was performed [41]. The whole-body dose was shown to increase with increasing administered activities per kg. While there was a significant correlation (r = 0.75; *p* < 0.001), there was appreciable variation, as although the mean whole-body dose was 0.23 mGy/MBq, the range was 0.071–0.43 mGy/MBq. The measured tumour dose also varied widely in this study (the median was 49 Gy), but the range covered an order of magnitude—26–378 Gy.

Historically, dosimetry has often not been performed, as it has not been perceived as being helpful. However, to personalise treatment and optimise outcomes, there is a lot of advantage to be gained from the routine use of dosimetry, and there is now a legal requirement to undertake this [42,43]. The art of molecular radiotherapy is intended to increase the dose received by cancer deposits so as to maximise the tumour control probability, while keeping organ at risk doses within the tolerance to minimise toxicity. There now follows some examples of how this may be achieved.

In a trial of ^177^Lutetium DOTATATE therapy for neuroblastoma, there was a need to keep renal doses below the cumulative tolerance dose of 23 Gy. For the following four courses of treatment, the renal radiation dose was only 16.5 Gy (range 9.5 to 21.5 Gy). These values represent the median and range of the cumulative mean renal radiation dose for each patient. This means that if a schedule that was prescribed to take the dose up to, but not to exceed, renal tolerance had been used, significantly more treatment could have been given, with the potential for better outcomes [22].

It is possible to personalise treatment and standardise the whole-body dose. For example, treatment may be prescribed to a desired total whole-body dose by delivering a dosimetry-adapted activity divided between two administrations. First, an initial weight-based activity is given, and the resulting whole-body dose is measured. Based on the dose received from this first administration, a second activity calculated to give a desired total whole-body dose is then administered. This results in a high degree of accuracy—the actual measured cumulative whole-body dose from the two fractions is close to the desired dose [44]. However, even when the whole-body dose is standardised, the tumour dose can still vary significantly, depending on the avidity of the tumour for ^131^I mIBG, and the duration of its retention [45]. In this paper, with a standardised cumulative whole-body dose of 4 Gy, the tumour dose varied from 10 to 103 Gy. As the whole-body dose may correlate with toxicity, and as administered activity may not correlate with whole-body dose received, it may help to prevent toxicity if, rather than using a standard administered activity, treatments are prescribed to a desired whole-body dose [46].

While absorbed doses can be measured after administration, it is possible to use the therapy agent’s theragnostic partner to predict the dose in advance. So, considering the theragnostic pairs of radiolabelled mIBG, a tracer dose of ^131^I mIBG may be given, followed by serial scans to estimate what the likely doses following ^131^I mIBG therapy might be. This technique was used to prevent excessive normal tissue toxicity in one escalated administered activity trial [41]. A new option arises when mIBG is labelled with the positron emitter ^124^I. Serial imaging with ^124^I mIBG PET CT has been used prior to ^131^I mIBG therapy to predict whole-body, organ and tumour absorbed radiation doses [47,48,49].

**Figure 4:**
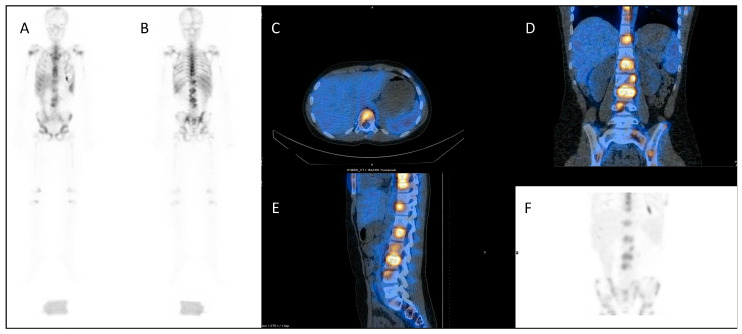
Dosimetry imaging with ^111^In-labelled anti-CD66 monoclonal antibody in a patient with relapsed leukaemia prior to ^90^Y anti-CD66 monoclonal therapy in an early phase trial [50]. (**A**) Anterior and (**B**) posterior whole-body planar scintigraphy views; (**C**) axial, (**D**) coronal and (**E**) sagittal fused SPECT CT views; and (**F**) SPECT maximum intensity projection image. These demonstrate avid uptake in areas of involved bone marrow.

A similar approach is used with serial ^111^In-labelled anti-CD66 monoclonal antibody SPECT (single photon emission computed tomography) CT imaging (Figure 4) for dosimetry prior to ^90^Y anti-CD66 monoclonal antibody therapy, as an alternative to total body irradiation as conditioning prior to allogeneic bone marrow transplantation for relapsed leukaemia, to ensure that organ at risk doses are not exceeded [50].

It is clear that there is considerable scope for dosimetry to personalise the approach to molecular radiotherapy, and this should be a key feature of future trials.

## 4. Response Assessment

Response assessment in oncology is often focused on the assessment of tumour size as shown on CT or magnetic resonance imaging. This process is typically formalised in clinical trials by the use of a standardised system such as RECIST (response evaluation criteria in solid tumors) [51]. However, in molecular radiotherapy the theragnostic pairs facilitate the use of molecular imaging, which may be more useful in personalising treatment.

For example, although in the treatment of metastatic neuroendocrine cancers a fixed number of courses of ^177^Lu DOTATATE is usually administered [2], in the treatment of metastatic thyroid cancer, personalisation is achieved through response-adapted therapy. Instead of deciding at the outset on a certain number of courses of ^131^I therapy, the response to one course is judged on the appearances of the scans following the next course, in conjunction with other factors such as the stimulated thyroglobulin level. Treatment continues until remission is achieved, or the disease stabilises.

In neuroblastoma, the semi-quantitative scoring of ^123^I mIBG images [15] forms the basis of the international response criteria [52]. As there is evidence that a poor response to initial chemotherapy, as demonstrated on ^123^I mIBG scintigraphy, correlates with a poor outcome, this is a factor in determining eligibility for VERITAS, a clinical trial to evaluate the role of ^131^I mIBG therapy in metastatic high-risk neuroblastoma showing a poor response to induction chemotherapy [53].

**Figure 5:**
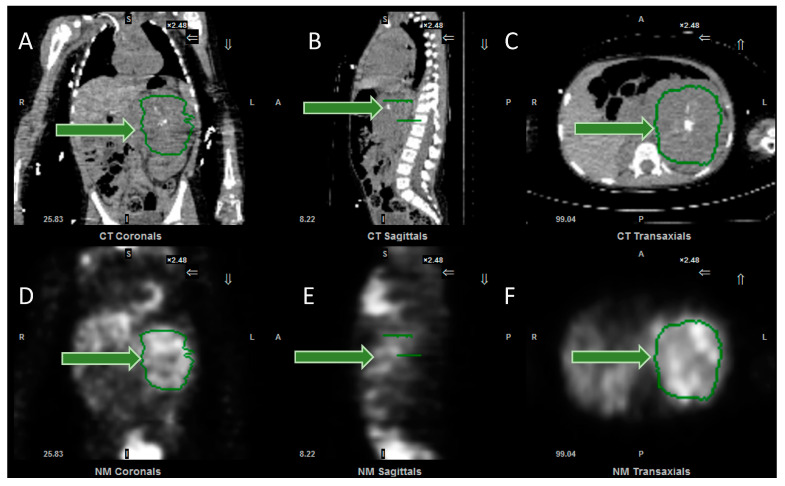
Images from the software to measure SPECT SUV (standardised uptake values). (**A**) Coronal, (**B**) sagittal and (**C**) axial CT images, together with (**D**) coronal, (**E**) sagittal and (**F**) axial SPECT images, demonstrating the tumour, outlined in green (arrowed). Following input of data, including administered activity and organ delineation, the system will calculate the minimum, maximum and mean (standard deviation) of the SUV, expressed as g/mL. Same patient as in Figure 1.

There is the prospect that in due course these semi-quantitative scores will be complemented or possibly even replaced by truly quantitative imaging. Quantitative PET CT imaging has been available for some time, with the measurement of various parameters, including standardised uptake values, metabolic active tumor volumes and total lesion glycolysis [54]. Now, software has been developed which makes it possible for SUV (standardised uptake values) to be objectively measured on SPECT CT scans (Figure 5), removing the need for more subjective image interpretation. Further research is required to determine the clinical value of serial measurements of this sort, and whether they enable greater personalisation of treatment.

## 5. Individualised Decision Making and Communication

Medicine is about caring for people, not simply treating disease. In molecular radiotherapy, practical and psycho-social considerations need to be taken into account for safe and effective personalised service delivery. Many different professional groups and disciplines need to work together to support a complex molecular radiotherapy service, including radiographers, physicists, nurses, pharmacists, and doctors from a range of specialties, including nuclear medicine, clinical, medical and paediatric oncology, surgery and endocrinology. Colleagues at other hospitals responsible for referring patients and sharing care are also involved. Excellent communication between these individuals is essential for safe and effective team working. This cannot be taken for granted, but requires a sustained and concerted approach.

HNAs (holistic needs assessments) are employed for multiple different cancer sites to ensure effective communication and information sharing between health professionals and the patient, leading to improved personalised quality care. HNAs are routinely employed for all patients receiving molecular radiotherapy prior to their treatment, to focus not only on the patient’s medical needs, but also to provide an in-depth assessment of the individual to inform the appropriate planning and implementation of treatment, thus improving patient experience [55]. The HNAs gather information regarding religious beliefs, psychological and social worries, support mechanisms for patients or families, preferred coping strategies, current stressors such as financial concerns or worries during their cancer experience, and any molecular radiotherapy-specific treatment-related concerns. The information received is presented at several MDT (multi-disciplinary team) meetings which include thyroid, paediatric, molecular radiotherapy and psycho-social team meetings. The specialist molecular radiotherapy team therefore acts as the main connection between the patient and the services through these MDT meetings, representing and defending vulnerable patient situations, patient safety, ensuring continuity of care and voicing these concerns to the wider team. This multi-disciplinary and multi-professional approach ensures that the best possible decisions are made regarding treatment and follow up care. Due to the complexity of medical needs, together with radiation protection principles, it is common for molecular radiotherapy patients to be discussed multiple times in the same MDT to allow for the best preparation and care for their stay on admission.

The molecular radiotherapy team is involved in each stage of the pathway, acting as the key contacts, and providing continuity of care with a healthcare professional that the patient knows and trusts. Management of the patients’ expectations of molecular radiotherapy from the outset after their initial referral cannot be underestimated, as patients often have pre-conceived ideas and misconceptions associated with radiation protection and isolation. To achieve realistic expectations, the patients have the opportunity to visit the specialist suites and ward where they will stay in advance of their treatment, so as to familiarise themselves with the surroundings. The facilities have been adapted for both adult and paediatric molecular radiotherapy patients, with a wide range of entertainment facilities on offer which are age appropriate dependent on the patient. Often, patients feel reassured and comforted by prior visits to the ward and special suites, improving the patient experience and reducing their anxiety prior to admission.

Due to the limited number and the geography of molecular radiotherapy services, and especially to access treatments and trials which are not widely available, patients often travel long distances to receive their care. After a thorough practical and logistical evaluation of the individual’s and family’s needs, a personalised approach to the organisation of outpatient clinical reviews (or other investigations) with diagnostic nuclear medicine imaging can ensure that these procedures coincide on the same day; this helps to minimise journeys to the hospital, thus alleviating the travel costs, absences from work and other family practicalities made difficult by hospital attendance. The use of accommodation nearby allows for the co-ordination of a series of investigations in a single return trip. Accommodation can also provide the comforter and carer a space for respite when their loved ones are receiving treatment, with necessary amenities for washing or cooking, for example.

For inpatient care of children, or adults with special needs such as physical disabilities or learning difficulties, appropriately selected non-pregnant adults, typically parents, may be legally designated as comforters and carers. This requires the provision of written information, and the training of the individuals in how to minimise their own personal radiation exposure while attending to the needs of the patient. The training involves guidance in the use of personal protective equipment, such as gloves, aprons and overshoes, to reduce the risk of contamination by radioactive body fluids, and an explanation of how increasing the distance from the patient, reducing close contact time, and the positioning of barriers will all help to reduce radiation exposure. Written informed consent is then received. When the correct procedure is followed, in-hospital radiation exposure is minimal for the majority of comforters and carers [56].

For any patient receiving molecular radiotherapy, their home circumstances will invariably affect the point in time after their treatment administration at which they can be discharged from hospital. This is evaluated prior to their admission by the molecular radiotherapy team using a radiation risk assessment.

For patients responsible for the care of a child under the age of 18 years old, or with a pregnant woman at home, the level of their residual activity needs to be lower. This results in an increased length of stay on admission, but mitigates the risk of ionising radiation exposure when they are discharged. Similar restrictions would also be placed on paediatric patients with young siblings at home. In contrast, a fit and capable patient living alone can be discharged at a much higher residual activity, and the patient is given personalised restrictions based on their needs and circumstances (as well as the dates on which these restrictions can be lifted). Administrations in an outpatient setting can be explored for patients meeting defined eligibility criteria relating to their home circumstances to minimise inpatient admission. Using the outpatient setting may improve patient experience by reducing disruptions to individuals and their families receiving treatment, and for some patients this may be a more suitable approach, for example those with increased anxiety or claustrophobia.

Follow-up arrangements can also be personalised according to need, with some investigations being arranged close to the patient’s home in the community or at the referring hospital, to minimise the need for travel to a tertiary centre.

## 6. Conclusions

To improve patient safety, clinical outcomes and the patient experience, it is important that the practice of molecular radiotherapy moves away from the empirical, standardised treatment protocols historically employed. Just because an intervention often gives reasonably satisfactory outcomes is no grounds for complacency. Results can be optimised through the thoughtful and evidence-based use of theragnostics. A scientific approach will individualise care, based on a number of factors including quantitative imaging to support treatment and administered activity selection, dosimetry to facilitate dose escalation within the parameters of organ at risk tolerance, response assessment to judge efficacy and guide further treatment, and personalisation based on human factors and good communication. The state of the art is not at present as good as it can be, and further clinical trials are needed to refine treatment schedules, as is basic science research to look at combination treatments with other agents, and to develop new theragnostic pairs for a wider range of diseases.

## Figures and Tables

**Table 1:** Examples of pairs of theragnostic radiopharmaceuticals.

Disease to be Treated	Molecular Imaging Radiopharmaceutical	Molecular Radiotherapy Radiopharmaceutical
Differentiated thyroid cancer	^123^I sodium iodide	^131^I sodium iodide
Neuroblastoma	^123^I mIBG ^1^	^131^I mIBG
Neuroendocrine cancers	^68^Ga DOTATATE ^2,3^ ^111^In Pentetreotide ^4^	^177^Lu DOTATATE ^90^Y DOTATOC ^5^
	^85^Sr strontium chloride	^89^Sr strontium chloride
Metastatic prostate cancer	^99m^Tc HDP ^6^	^153^Sm lexidronam ^186^Re etidronate ^223^Ra radium dichloride
	^68^Ga PMSA ^7^	^177^Lu PMSA
Acute leukaemia	^111^In anti-CD66 monoclonal antibody	^90^Y anti-CD66 ^8^ monoclonal antibody

^1^*meta*-iodobenzylguanidine; ^2^ dodecane tetraacetic acid-octreotate; ^3^ for PET (positron emission tomography) imaging; ^4^ for gamma camera imaging; ^5^ Edotreotide; ^6^ hydroxymethylene diphosphonate; ^7^ prostate specific membrane antigen; ^8^ anti-cluster of differentiation 66.

**Table 2:** Typical basis for selection of administered activities and routine dosimetry performed.

Disease to be Treated	Molecular Radiotherapy Radiopharmaceutical	Dosimetry Notes
Differentiated thyroid cancer	^131^I sodium iodide	Routinely fixed administered activity. Two means of personalised dosimetry: the lesion/remnant disease-based method; and the bone marrow dose method
Neuroblastoma	^131^I mIBG	Administered activity scaled to weight or body surface area. Can be tailored to individual patients based on whole-body absorbed dose.
Neuroendocrine cancers	^177^Lu DOTATATE ^90^Y DOTATOC	Routinely fixed administered activity. ^177^Lu DOTATATE dosimetry can be calculated using the gamma emissions to obtain post therapy planar images; ^90^Y DOTATOC not routinely possible.
	^89^Sr strontium chloride	Fixed administered activity. Dosimetry not routinely performed
Metastatic prostate cancer	^153^Sm lexidronam ^186^Re etidronate ^223^Ra radium dichloride	^153^Sm lexidronam weight-based administered activity; ^186^Re etidronate fixed administered activity; no more detailed dosimetry routinely performed. ^223^Ra photons allow for post-treatment imaging. Low-dose pre-treatment administration also allow potential for greater dosimetric accuracy.
	^177^Lu PMSA	^68^Ga PSMA diagnostic imaging used for pre-therapeutic dosimetric calculation; ^177^Lu gamma emission allows dosimetric calculation for subsequent therapies.
Acute leukaemia	^90^Y anti-CD66 monoclonal antibody	^111^In labeled anti-CD66 monoclonal antibody single photon emission computed tomography used for dosimetric calculation prior to therapy to ensure bone marrow limit not exceeded.
